# *Exophiala campbellii* causing a subcutaneous palmar cyst in an otherwise healthy UK resident

**DOI:** 10.1016/j.mmcr.2020.07.004

**Published:** 2020-08-05

**Authors:** Andrew M. Borman, Mark Fraser, William Schilling, Gillian Jones, Robert Pearl, Christopher J. Linton, Elizabeth M. Johnson

**Affiliations:** a UK National Mycology Reference Laboratory, Public Health England, Science Quarter, Southmead Hospital, Bristol, BS10 5NB, United Kingdom; b Medical Research Council Centre for Medical Mycology (MRC CMM), University of Exeter, Exeter, EX4 4QD, United Kingdom; c Royal Sussex County Hospital, Brighton and Sussex University Hospitals NHS Trust, Eastern Road, Brighton, BN2 5BE, United Kingdom; d Queen Victoria Hospital NHS Foundation Trust, Holtye Road, East Grinstead, West Sussex, RH19 3DZ, United Kingdom

**Keywords:** *Exophiala* species, Subcutaneous cyst, Phaeohyphomycosis, Immunocompetent, Black yeast

## Abstract

*Exophiala* is a ubiquitous genus encompassing more than forty species, a number of which have been associated with superficial or systemic infections in humans, and other hot- or cold-blooded animals. Here we report a human case of subcutaneous mycotic cyst caused by *Exophiala campbellii*. To our knowledge, this is only the third reported human infection caused by *E. campbellii*, all three of which involved subcutaneous nodules in patients who had resided in the United Kingdom.

## 1 Introduction

The saprobic genus of “black yeast-like fungi” *Exophiala (Herpetotrichiellaceae)*, which has cosmopolitan distribution, contains many potential pathogens of warm and cold-blooded animals with the ability to cause infections in specific hosts determined at least in part by the ability to grow at body temperature [[1], [2], [3], [4], [5]]. Infections in humans range from colonisation of the respiratory tract in patients with cystic fibrosis [6,7], subdermal cysts and widespread phaeohyphomycosis [5,8] or eumycetoma [3], to fatal systemic and disseminated infections in both immunocompetent and immunocompromised hosts [3,[8], [9], [10]]. Cutaneous and subcutaneous *Exophiala* infections usually follow traumatic inoculation of fungal material after penetrating injuries with wood/plant material [[11], [12]], while the portal of entry for systemic infections is often less easily defined [1,3,8].

All members of the genus *Exophiala* share overlapping morphological features in culture, including dematiaceous (melanised hyphae) and conidia produced in wet masses from annellidic conidiophores [[1], [2], [3], [4], [5]]. This lack of morphological differentiation between species impedes accurate phenotypic identification, and molecular [3,5,8] or proteomic [5] approaches are essential for accurate identification and the delineation of the spectrum of clinically relevant *Exophiala* species. Using such approaches, we recently described a novel *Exophiala* species, *E. campbellii*, amongst a collection of historical clinical isolates stored in the UK National Collection of Pathogenic Fungi (NCPF) [5].

## 2 Case

The patient, a 68 year old female UK resident, presented in April 2019 with a subcutaneous cystic lesion on the right palm containing thick yellowish-brown fluid. The patient recalled a penetrating injury approximately at the site of the lesion, involving a thorny plant whilst on vacation in Fiji three years previously, and indicated that the lump had developed over the six months preceding her presentation.

**Fig. 1:**
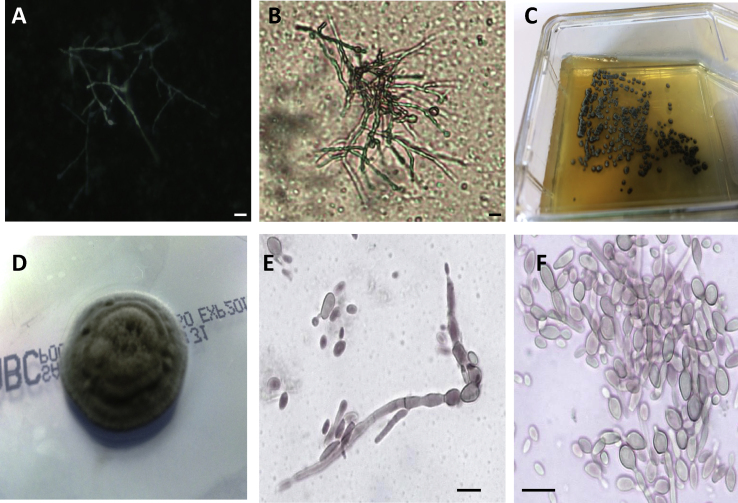
**Mycological examination of cyst fluid.** Direct microscopic examination of cyst fluid stained with KOH/calcofluor fluorescent enhancer and visualized under UV light (A) or examined under direct light (B). Note the dematiaceous fungal hyphae under direct light, and patchy staining with calcofluor due to fluorescence quenching in melanin-rich hyphal portions. Organism recovered from 10 μl of cyst fluid cultured on Sabouraud agar at 30 °C for 5 days (C) and appearance of a single colony sub-cultured on Sabouraud agar after 14 days at 30 °C (D). Panels E and F: microscopic appearance of tease mounts prepared from colonies incubated at 30 °C (E) and 37 °C (F). Scale bars = 10 μm.

The cyst, which appeared encapsulated, was excised surgically in its entirety. Microscopic examination of fluid from the cyst revealed large amounts of filamentous fungal elements (Fig. 1A), some of which resembled toruloid mycelia (xylohyphae), and portions of which were conspicuously dematiaceous in appearance (Fig. 1B). Culture of cyst fluid yielded pure growth of fungus at 30 and 37 °C (Fig. 1C), with faster growth at the lower temperature. Fungal colonies on Sabouraud agar were flat with raised, floccose, folded or umbilicate centres. Colonies were velvety, olivaceous-grey to brown with dark greenish-grey colony reverse (Fig. 1D). Microscopic examination of tease mounts prepared from fungal growth in culture at 30 °C (Fig. 1E) revealed regular pale brown, septate hyphae and scant budding cells, which were oval to ellipsoidal, light olivaceous-green and measured 2.5 by 3.5 μm. Budding cells predominated in cultures maintained at 37 °C (Fig. 1F), and often had a short annellated zone. Since colonial and microscopic appearance of the organism was consistent with members of the genus *Exophiala*, for which we have previously developed an extensive mass spectral database [5], the isolate was subjected to MALDI-TOF MS analyses. Mass spectral profiles matched those of *Exophiala campbellii* [5] in the MRL database with reasonable MeanLogScores (2.012). Formal confirmation of identity was achieved through PCR amplification and sequencing of portions of the 28S rRNA gene and the internal transcribed spacer region 1 (ITS) performed exactly as described previously [5]. The sequences generated were 100% identical to those corresponding to the type strain of *Exophiala campbellii*, isolate NCPF 2274 (EMBL accession numbers for the current case MN091927 and MN091928). The isolate from the current case is stored in metabolically inactive form in the NCPF with the unique identifier NCPF 7936. Antifungal susceptibility testing was performed on isolate NCPF 7936 using CLSI methodologies [13]. Minimum inhibitory concentrations (in mg/L) for amphotericin B, itraconazole, posaconazole and voriconazole were 0.5, 0.25, 0.125 and 0.125, respectively. Further confirmation that *E. campbellii* was the etiological agent was obtained by pan-fungal PCR amplification and sequencing of fungal genomic DNA extracted directly from cyst fluid, which was performed exactly as described previously [14]. Pan-fungal PCR was positive with primers targeting both the 28S rRNA gene and ITS1, and sequences generated from both amplicons again matched those of *E. campbellii* in the public synchronized EMBL database (data not shown). Since the surgical team was confident that the cyst had been completely excised, antifungal therapy was not initiated post-operatively.

## 3 Discussion

Cutaneous and subcutaneous infections with dematiaceous (melanised) fungi are typically classified as phaeohyphomycosis or chromoblastomycosis dependent on the tissue form of the fungus, with chromoblastomycosis characterised by the presence of sclerotic bodies instead of typical fungal hyphae [1]. Most previous cases of subcutaneous infection with *Exophiala* species reported hyphal tissue forms typical of phaeohyphomycosis, as was the case with the fungal elements visualized directly in the cyst fluid from the current case. *E. campbellii* was first described in 2016 [5], based on two historical isolates stored in a culture collection. Phylogenetic analyses placed *E. campbellii* in the *E. dermatitidis* clade, which contains most of the recognised human pathogenic species, and suggested that *E. campbellii* was most closely related to *E. oligosperma* [5]. *E. oligosperma* is a rare, but known cause of subcutaneous fungal infections, having previously been reported from solitary mycotic cysts and more disseminated subcutaneous phaeohyphomycosis in both immunocompetent and compromised patients [[15], [16], [17], [18], [19]].

The only two previous known isolates of *E. campbellii* were also recovered from subcutaneous human infections [5] (see below), and to our knowledge the species has never been recovered from the environment. The type strain was isolated from a foot ganglion of an otherwise healthy female living in Germany in 1981, and the second isolate was cultured from biopsy tissue from a UK patient with chest nodules in 1984. Interestingly, although the original case involved a patient in Germany, she was the British wife of a UK soldier stationed overseas and had previously resided in the UK. The current case also involved a UK resident, raising the possibility that *E. campbellii* might be geographically restricted, as has been proposed for distinct clonal populations of the neurotropic species *Exophiala dermatitidis* [20]. However, since the patient recalled a previous penetrating injury at the site of the lesion whilst visiting Fiji, it is also possible that the infection was acquired overseas. Regardless of the exact worldwide distribution of the organism, the current report of a subcutaneous mycosis caused by *E. campbellii*, more than 3 decades after the initial two cases, confirms that this species should be added to the growing list of human fungal pathogens in the genus *Exophiala*. Finally, since the cyst had been completely excised in the current case, no antifungal therapy was initiated. However, the antifungal susceptibility profile of the current isolate, which agreed well with the MICs reported for the two previous isolates of *E. campbellii* [5], would suggest that itraconazole, posaconazole or voriconazole would each be appropriate therapeutic choices.

## Consent

Written informed consent was obtained from the patient or legal guardian(s) for publication of this case report and accompanying images. A copy of the written consent is available for review by the Editor-in-Chief of this journal on request.

## Funding

None.

## Declaration of competing interest

None to declare.
